# Gene Expression Profiling of Development and Anthocyanin Accumulation in Kiwifruit (*Actinidia chinensis*) Based on Transcriptome Sequencing

**DOI:** 10.1371/journal.pone.0136439

**Published:** 2015-08-24

**Authors:** Wenbin Li, Yifei Liu, Shaohua Zeng, Gong Xiao, Gan Wang, Ying Wang, Ming Peng, Hongwen Huang

**Affiliations:** 1 Key Laboratory of Plant Germplasm Enhancement and Specialty Agriculture, Wuhan Botanical Garden, Chinese Academy of Sciences, Wuhan, Hubei, China; 2 Institute of Tropical Bioscience and Biotechnology, Chinese Academy of Tropical Agricultural Sciences, Haikou, Hainan, China; 3 Key Laboratory of Plant Resources Conservation and Sustainable Utilization, South China Botanical Garden, Chinese Academy of Sciences, Guangzhou, Guangdong, China; 4 Key Laboratory of Biology and Genetic Resources of Tropical Crops, Ministry of Agriculture, P. R. China, Haikou, China; 5 University of Chinese Academy of Sciences, Beijing, China; NARO Institute of Fruit Tree Science, JAPAN

## Abstract

Red-fleshed kiwifruit (*Actinidia chinensis* Planch. ‘Hongyang’) is a promising commercial cultivar due to its nutritious value and unique flesh color, derived from vitamin C and anthocyanins. In this study, we obtained transcriptome data of ‘Hongyang’ from seven developmental stages using Illumina sequencing. We mapped 39–54 million reads to the recently sequenced kiwifruit genome and other databases to define gene structure, to analyze alternative splicing, and to quantify gene transcript abundance at different developmental stages. The transcript profiles throughout red kiwifruit development were constructed and analyzed, with a focus on the biosynthesis and metabolism of compounds such as phytohormones, sugars, starch and L-ascorbic acid, which are indispensable for the development and formation of quality fruit. Candidate genes for these pathways were identified through MapMan and phylogenetic analysis. The transcript levels of genes involved in sucrose and starch metabolism were consistent with the change in soluble sugar and starch content throughout kiwifruit development. The metabolism of L-ascorbic acid was very active, primarily through the L-galactose pathway. The genes responsible for the accumulation of anthocyanin in red kiwifruit were identified, and their expression levels were investigated during kiwifruit development. This survey of gene expression during kiwifruit development paves the way for further investigation of the development of this uniquely colored and nutritious fruit and reveals which factors are needed for high quality fruit formation. This transcriptome data and its analysis will be useful for improving kiwifruit genome annotation, for basic fruit molecular biology research, and for kiwifruit breeding and improvement.

## Introduction

Kiwifruit (*Actinidia* Lindl.) is a sought after fresh fruit in the marketplace due to its unique flavor and nutritional components, such as high vitamin C, amino acid and mineral contents [1,2]. Kiwifruit cultivars are mostly developed from the *A*. *chinensis* Planch species complex, including the two varieties var. *chinensis* and var. *deliciosa* [2]. A large germplasm resource containing varied characteristics, including flesh of different colors, is found in China [3]. Over the past decades, green-fleshed kiwifruit cultivars have dominated the worldwide commercial market. However, in recent years consumers have shown interest in fruits with high levels of anthocyanins, due to their antioxidant and health-promoting properties [4]. Interest in the international market is shifting from green-fleshed to yellow- or red-fleshed kiwifruit cultivars. In recent years, the red-fleshed cultivar *A*. *chinensis* cv ‘Hongyang’ was developed commercially in China. By 2011, it accounted for about 15.3% of the total kiwifruit plantings in China, and sold at a higher price than the green variety at market [2]. ‘Hongyang’ is popular not only because of its unique anthocyanin accumulation, but also due to its higher sugar (of 8.97–13.69% of fresh weight) and vitamin C (of 135–250 mg 100 g fresh weight^-1^) contents [2,5]. In addition, matures early, at 140 days, which was particularly preferred by growers [6]. Overall, ‘Hongyang’ is a promising cultivar.

Fruit development and ripening are necessary for fruit-bearing plants to complete their life cycle. Although transcript profile during fruit development are available for other fleshy fruits, such as Chinese bayberry (*Myrica rubra*) [7], pineapple [8], and tomato[9], our understanding of the mechanism controlling development of quality kiwifruit remains quite limited. For instance, candidate genes related to basic development and phytohormone production or to production of important characteristics such as sweetness or high vitamin C and anthocyanin content have not been explored in depth. The recently published ‘Hongyang’ genome makes this type of deep molecular research possible [10]. Thus, in the present work, RNA samples from seven different developmental stages of the fruit-forming ovary and fleshy pericarp were sequenced. During analysis of the transcriptome data, we focused on candidate genes for phytohormone biosynthesis and signal transduction, sugar and starch biosynthesis and breakdown, L-ascorbic acid biosynthesis and metabolism, and flavonoid biosynthesis and regulation.

Genes involved in anthocyanin biosynthesis and regulation have been discovered and studied in many fruits, such as *MdbHLH3* and *MdUFGT* in apple [11,12], *LcUFGT* in litchi [13], *VvF3’5’H* in grape [14,15], *MYB10*.*1*, *MYB10*.*3* and *bHLH3* in peach[16], *PcMYB* 10 in pear [17,18], and *MYB110a* and *F3GT1* in kiwifruit[19,20]. Anthocyanin biosynthesis is a branch of the flavonoid pathway and is also altered by the presence or absence of upstream genes. Thus we analyzed the transcript levels of all the genes involved in both flavonoid and anthocyanin biosynthesis in order to demonstrate the dynamics of anthocyanin biosynthesis and accumulation in ‘Hongyang’.

In addition, transcriptome or RNA-seq data is the strong technique to identify novel transcripts (NTs) and alternative splicing (AS) in plants, such as Arabidopsis and rice [21,22]. NTs that do not fall within genome annotation should enlarge the extent of genome and AS also showed tissue specificity [23]. We also analyzed the NTs and AS in different developmental stages of kiwifruit to find the developmental specificity of NT and AS.

The transcriptome should also be used to explore genes committed to other characteristics such as the low organic acid, early maturity and enjoyable flavor.

## Materials and Methods

### Plant materials

‘Hongyang’ (*A*. *chinensis*) red kiwifruit at seven different stages [S1 = 7, S2 = 70, S3 = 90, S4 = 100, S5 = 110, S6 = 120 and S7 = 140 days after anthesis (DAA)] were collected from the Wuhan Botanical Garden, Hubei Province in 2012. Transcriptome sequencing was conducted on either the whole ovary (S1) or mesocarp flesh, with seeds removed from S2—S7.

### Library preparation for transcriptome analysis

Total RNA was isolated using a modified CTAB method [24]. RNA was treated with RNase-free DNase I (Ferments Inc.) for 30 minutes at 37°C. RNA integrity was determined using the Agilent 2100 Bioanalyzer with a minimum integrity number (RIN) of 7.3 after denaturing at 70°C for 2 min. A total of seven cDNA libraries of fruit samples (S1-S7) were constructed for RNA-Seq. Every library was composed of three equally mixed mRNA samples from three different fruits at the same stage.

### Illumina sequencing and assembly

All cDNA libraries were sequenced using an illumina 2000 by the Beijing Genome Institute (BGI) (Shenzhen, China) according to the protocols for RNA-Seq [25]. The raw reads were cleaned by removing adaptor sequences and low quality reads with ambiguous ‘N’ base calls. Clean reads were aligned to the kiwifruit reference genome database. (http://bioinfo.bti.cornell.edu/cgi-bin/kiwi/home.cgi) through *SOAPaligner*/*SOAP2* with default parameters (allowing up to 5 mismatches) [26]. The function of genes was annotated using BLASTx with an E-value threshold of 10^−5^ to the NCBI nr, Swiss-Prot, KEGG, COG, and Kiwifruit Genome ([10] http://bioinfo.bti.cornell.edu/kiwi) databases. The transcriptome datasets are available at the NCBI Sequence Read Archive (SRA) with the accession number SRR926770. The Transcriptome Shotgun Assembly has been deposited at DDBJ/EMBL/GenBank under the accession numbers GALZ00000000, GAMA00000000 and GAMB00000000.

Gene structure refinement and novel transcripts were constructed using *Cufflink* [27] and the alternative splicing was analyzed through *Tophat* [28]. To valuate the possible coding functions of novel transcripts, we used a Support Vector Machine-based classifier, named Coding Potential Calculator (CPC http://cpc.cbi.pku.edu.cn/ [29]).

### Analysis of gene expression level

The gene expression levels were calculated using the Reads Per Kilobase transcriptome per Million mapped reads (RPKM) method [30].

The differentially expressed genes (DEGs) were screened with the threshold of FDR <0.001 and |Log_2_Ratio| ≥1 [Ratio = (S_n+1_/S_n_) where S_n_ represents the RPKM value of genes at different developmental stages (n, 1 to 6)] between two neighboring developmental stages [31,32].

### Metabolic pathways analysis

Metabolic pathways analysis was carried out via MapMan software (version 3.5.1R2) (http://mapman.gabipd.org/) [33] based on top hits to the Kiwifruit Genome Database.

Hierarchical cluster analysis of the expression levels of genes committed to all pathways, including phytohormone, sucrose, starch, L-ascorbic acid and anthocyanin metabolism, was conducted via MultiExperiment Viewer (MeV) (version 4.8.1) from the TM4 suite (http://www.tm4.org/mev.html) [34].

### Determination of phytohormone, total soluble sugar, starch and L-ascorbic acid levels

To estimate the abscisic acid (ABA), cytokinin (CK), auxin (AUX) and gibberellins (GA) levels, fresh flesh (5 g) from each stage was ground in liquid nitrogen and treated with 10 ml cold 80% (v:v) methanol through a modified method [35]. Sample was analyzed by reversed-phase HPLC (Agilent 1260, USA). Separation was achieved with a 4.6 × 150 mm i.d., 3.5 μm, C18 column (Waters, USA) at 35°C using a methanol: acetonitrile: phosphate buffer (0.01M, pH3.5) = 15:20:65 (v/v /v). Monitoring for AUX, ABA and GA was at 254 nm and for CK was at 265 nm. The chromatographic peaks were achieved by comparing retention times with authentic standards of AUX, ABA, CK and GA (Sigma, USA) and identified by a combination of liquid chromatography (Ultimate 3000, Dionex, USA)-tandem mass spectrometry (AmaZon SL, Bruker Daltonics, Germany) (LC-MS).

Total soluble sugar was measured through anthrone-H_2_SO_4_ colorimetry [36]. Starch content was estimated by the phenol-sulfuric method [37]. A glucose standard solution was used to prepare the working standard. An ultraviolet spectrophotometer (Libra S22, biochrom) was used for measurement. The total soluble solids (TSS) were measured by potable digital refractometer (PAL-1, ATAGO, Japan).

The content of L-ascorbic acid (Vitamin C) was detected by HPLC [38].

### Anthocyanin extraction and evaluation by HPLC

Kiwifruit samples [5 g fresh weight] from S1 to S7 were ground in liquid nitrogen before anthocyanin extraction using 5:1 (v/w) ethanol/H_2_O/acetic acid (80:20:1 v/v/v) in an Ultra-Turrax homogenizer for 30 minutes. Homogenates were held for 24 hours at 4°C in the dark. The supernatant was then collected, filtered with 0.45 μm filter paper, and retained for HPLC analysis [39]. Extracts were analyzed by reversed-phase HPLC using an Alliance 2695 instrument equipped with a 996 photodiode array detector (Waters, USA). Separation was achieved with a 150 ×3.9 mm i.d., 4 μm, C18 Aqua column (Nova-pak, Waters), and a binary solvent system of (A) 1% aqueous formic acid (v/v) and (B) acetonitrile. Anthocyanin was monitored at 530nm, and chromatographic peaks were identified by comparison with authentic standards of cyaniding 3-O-galactoside (Chromadex USA, CAS No. 27661-36-5) and cyaniding 3-O-glucoside (WAKO, Jap, CAS No. 7084-24-4).

### Phylogenetic analysis of kiwifruit genes

Neighbor-joining phylogenetic trees were constructed using MEGA software version 5.0 [40]. Trees were constructed with the kiwifruit protein sequences and sequences from functionally characterized proteins of the same class. The tree construction parameters and accession numbers are provided in the figure legends.

### Quantitative real-time polymerase chain reaction (qRT-PCR) analysis

RNA for transcriptome sequencing was used for qRT-PCR analysis. The PCR mixture contained 1 μl of the cDNA template, 10 μl of 2× mixture (DRR041A, Takara), and 0.2 μM of the forward and reverse primers for each gene. Reactions were run on an Applied Biosystems StepOne Real-Time PCR system with a 96-well plate (Applied Biosystems, Foster City, CA, USA). The conditions for each PCR reaction were as follows: 95°C for 30 s, followed by 40 cycles of 5 s at 95°C, 20 s at 58–62°C depending on primer TM, and 20 s at 72°C. At the end of each experiment, a melt-curve analysis was performed using the default parameters (15 s at 95°C, 1 m at 55°C-95°C in 0.3°C increments, 15 s at 95°C). *Actin* (GI:149938963) was used as the reference gene.

## Results

### ‘Hongyang’ kiwifruit development

**Fig 1:**
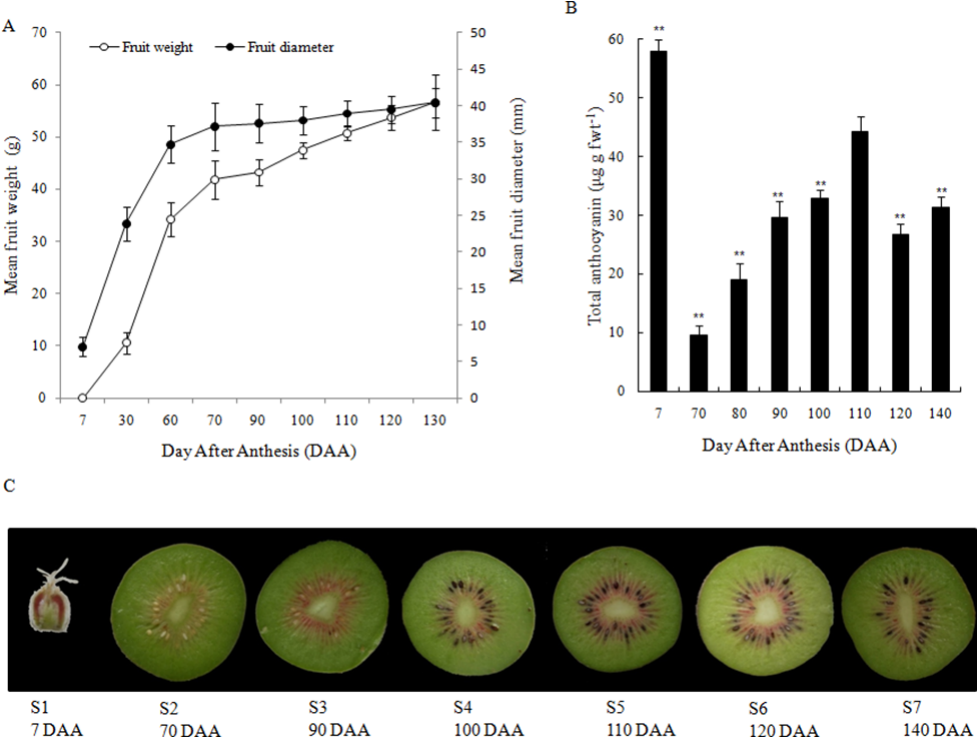
Developmental stages of *A*. *chinensis* cv. ‘Hongyang’ used in deep transcriptome analysis. (A) Mean fruit weight and diameter throughout development. Data are means ± (n = 10–12). (B) The content of anthocyanins in ‘Hongyang’ during fruit development. Values are means of five replicates. (C) Bisected fruit across seven developmental stages (S1-S7) and corresponding DAA. The error bars represent SE. DAA, day after anthesis. Asterisks indicate significantly different (*P<0.05, **P<0.01) from value at 110 DAA (S5).

‘Hongyang’ fruits were collected at seven developmental stages, from fruit-forming ovary to ripening over 140 days after anthesis (DAA). Morphological characters were measured based on the changes of fruit size and the color of fruit flesh. The kiwifruit experienced the fastest growth stage from 30 to 70 DAA, and then grew slowly until ripening at a 55–60 mm diameter (Fig 1A). The highest concentrations of anthocyanin occurred during fruit formation (5–10 DAA, S1), but disappeared completely from 30 to 70 DAA. During the initial ‘expansion’ phase, young fruits were hard and green, but anthocyanin accumulation started at 90 DAA and continued until ripening, with the levels fluctuating at the late stages (Fig 1B). The deepest red color in pericarp flesh visually appeared from 100 to 110 DAA. The measurable anthocyanin content was the highest at 110 DAA, after which it decreased (Fig 1B). These 7 stages were used for fruit development and transcriptome sequencing analysis (Fig 1C).

### Kiwifruit transcriptome profiling

#### Overview of transcriptome quality and coverage

Analysis of sequencing and alignment quality indicated that the RNA-seq data reads in the seven developmental stages were evenly distributed on the reference Kiwifruit Genome (Fig A in S1 File). In each fruit developmental stage, 39% to 49% of the expressed genes had an RNA-seq coverage rate of 90–100% (Fig B in S1 File). Across the stages, an average of 86.8% of the reads mapped to the reference genome, and 42.6% of reads mapped to annotated genes (Table 1). A total of 32,536 genes were expressed within the fruit transcriptome, most of which were expressed in S1 (30,988) and the expression levels of genes were shown (Table A in S5 File). The highest number of AS events (24,839 events) occurred at S1 and the least (9,993 events) at S7. The ratio of the AS genes to all expressed genes was 50.63% in S1 (Table 1). The lower occurrence of AS during S2 to S7 (average of 30.1%) may be due to the fact that RNA was extracted only from kiwifruit flesh without seeds or peel, compared to the full fruit sample of S1. Intron retention accounted for the most alternatively spliced transcripts, which occurred at similar rates in Arabidopsis and maize [22,23] (Table B in S5 File).

**Table 1:** Analysis of RNA-seq data from kiwifruit flesh.

	Category	S1	S2	S3	S4	S5	S6	S7
RNA-seq data	Total clean reads	63940336	46494814	55622726	60252800	62153504	63429812	52188414
	Genome map reads (rate)^a^	54521996(85.27%)	39937851(85.90%)	48511408(87.22%)	51613020(85.66%)	54227442(87.25%)	56568728(89.18%)	45509887(87.20%)
	Gene map reads (rate)^a^	30193986(47.22%)	21179496(45.55%)	23100917(41.53%)	29032621(48.18%)	25264646(40.65%)	22030439(34.73%)	21046928(40.33%)
Analysis	Expressed Gene	30988	27286	27138	27944	26943	26639	25006
	Alternative Splicing event (Gene)^b^	24839(15692)	12061(8089)	11990(7747)	16574(10614)	13256(8478)	14119(8984)	9993(6193)
	Novel transcripts (Gene)^c^	6928(4578)	4631(3244)	4894(3304)	5300(3633)	5214(3494)	4923(3329)	4538(3012)

^a^ Reads were aligned to the Kiwifruit Genome by SOAPaligner/SOAP2 and TopHat.

^b^ Detected by TopHat.

^c^ Assembled by Cufflink

Identification of candidate NTs will contribute to the gene sequences available in the public Kiwifruit Genome. NT exon analysis showed that >30% of the NTs had 2 exons and <7% had 10 or more exons and more than 70% of candidate NTs had protein coding potential (Table B in S5 File). Furthermore, more than 9,000 genes sequences have been extended from 5’ or 3’ end compared to the annotations in the whole genome sequence database. For example, achn013171 (encoding zeaxanthin epoxidase in ABA biosynthesis), achn023421 (encoding an auxin response factor), and achn024141 (Sucrose synthase) were extended 143, 237, and 213 bp from their 3’ ends, respectively (Table B in S5 File). This data helped us to determine their sequence and the calculation of expression levels more accurately.

#### Gene expression profiling during kiwifruit development

We used qRT-PCR to validate the expression levels of more than 80 genes studied in the paper. Except that some genes could not be amplified through qRT-PCR due to unmatched primers, there was a high correlation (R^2^ = 0.9188) between RNA-seq and qRT-PCR for 74 genes, implying that the transcript value nearly reflect the true level of expression levels of all expressed genes (Table C in S5 File).

Differentially expressed genes (DEGs) were used to explore the transcript profiles of the different stages. The ovary (S1) had the highest number of DEGs compared to its following stage, S2: 8926 genes were more highly expressed in S1 than in S2, while 1958 genes were more highly expressed in S2 than in S1 (Table 2). For each developmental transition from S1 to S4, there was more up-regulated DEGs than down-regulated, implying that fruit was undergoing substantial transcriptional changes during growth. For each transition from S4 to S6, there were more down-regulated genes, suggesting that cell fates were determined and that fruit was only subject to expansion once black seeds appeared.

**Table 2:** The number of DEGs between two neighboring stages of ‘Hongyang’ kiwifruit development.

DEGs	S2 vs S1	S3 vs S2	S4 vs S3	S5 vs S4	S6 vs S5	S7 vs S6
UP	1958	1623	2315	876	1293	1547
DOWN	8926	1399	1607	2566	3254	1481

DEGs with FDR <0.001 and |Log_2_Ratio| ≥1 (Ratio = (S_n+1_/S_n_) where S_n_ represents the RPKM value of genes at developmental stages (n) from 1 to 6).

**Fig 2:**
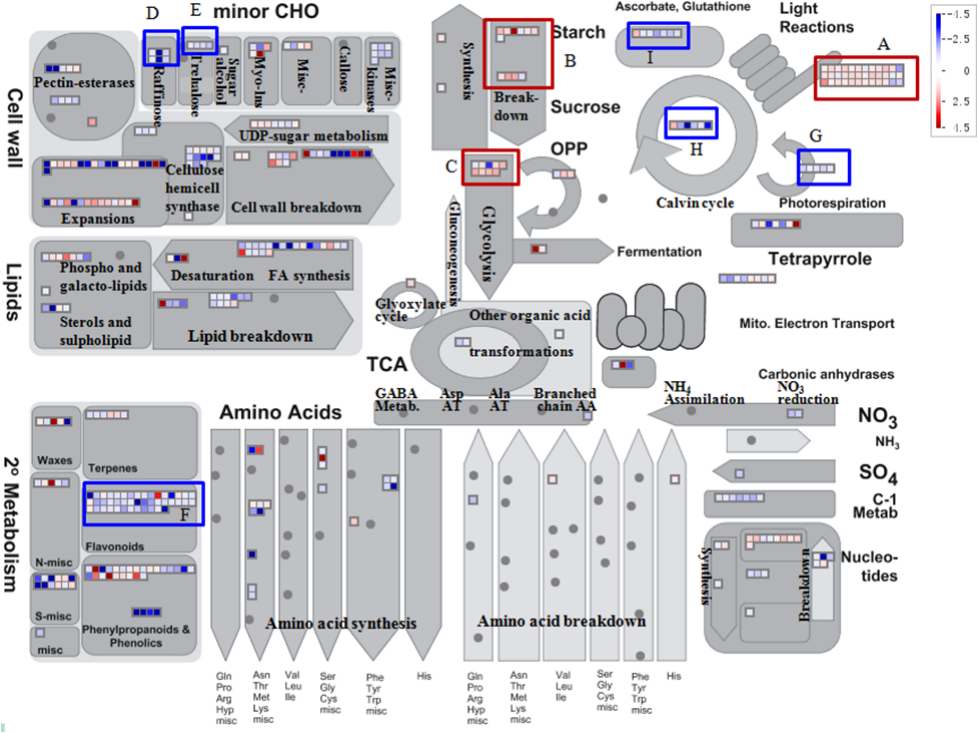
Metabolic overview of DEGs in *A*. *chinensis* ‘Hongyang’ at S5 compared to at S4. Genes with FDR <0.001 and |Log_2_Ratio| ≥1 (Ratio = S_5_/S_4_) were considered to be DEGs. Mapping: Kiwifruit Genome database, mapped: 3522 of 3214 data points, visible: 422 data points. The heat map ranges from -4.5 (blue; down-regulated) to 4.5 (red; up-regulated). Labels refer to genes in light reaction (A), starch and sucrose break down (B), glycolysis (C), raffinose (D), trehalose (E), flavonoids (F), photorespiration (G), calvin cycle (H), and ascorbate & glutathione (I).

MapMan software was used to assign genes to functional categories. S5 means an important line for kiwifruit development, after which kiwifruit development biologically slows down. The lowest number of un-regulated DEGs was between S4 and S5. Genes involved in light reactions, starch and sucrose degradation, and glycolysis were greatly up-regulated in S5, while most of genes related to the metabolism of raffinose, trehalose, ascorbate, calvin cycle, photorespiration and flavonoids were significantly down-regulated at S5 compared to at S4 (Fig 2). Genes committed to amino acid synthesis and break down were similarly expressed between S4 and S5. The highest number of DEGs was between S1 and S2. Comparison of S2 to S1, many of the DEGs encoding enzymes related to metabolism of cell wall, lipids, and glycolysis were greatly down-regulated. DEGs involved in secondary metabolism, such as photorespiration and terpenes, flavonoid and phenylpropanoid biosynthesis, also were expressed at the lower levels at S2 (Fig A in S2 File). At fruit ripening (S7), there was a balance of up- and down-regulated genes (Table 2), and most metabolic genes were greatly increased in S7 compared to S6 (Fig B in S2 File). For example, the light-harvesting chlorophyll a/b binding protein, the glyoxylate aminotransferase involved in photorespiration, the phosphoric diester hydrolase in lipid catabolism, and the glucose-1-phosphate adenylyltransferase related to starch biosynthesis were more highly expressed in the final fruit developmental stage (Fig B in S2 File). Most of DEGs involved in secondary metabolism were up-regulated with the maturation of kiwifruit at S7 including chalcone synthase (achn168721), chalcone isomerase (achn328591), flavonoid 3'-hydroxylase (achn194551), leucoanthocyanidin dioxygenase (achn361621 and achn002561), dihydroflavonol reductase (achn135311), and UDP-glucose: flavonoid 3-o-glucosyltransferase (achn209671), which together likely contributed to the higher content of anthocyanins at S7 compared to at S6. All DEGs were shown in Table D in S5 File.

### Secondary metabolism during kiwifruit development

#### Phytohormone content and gene expression

**Fig 3:**
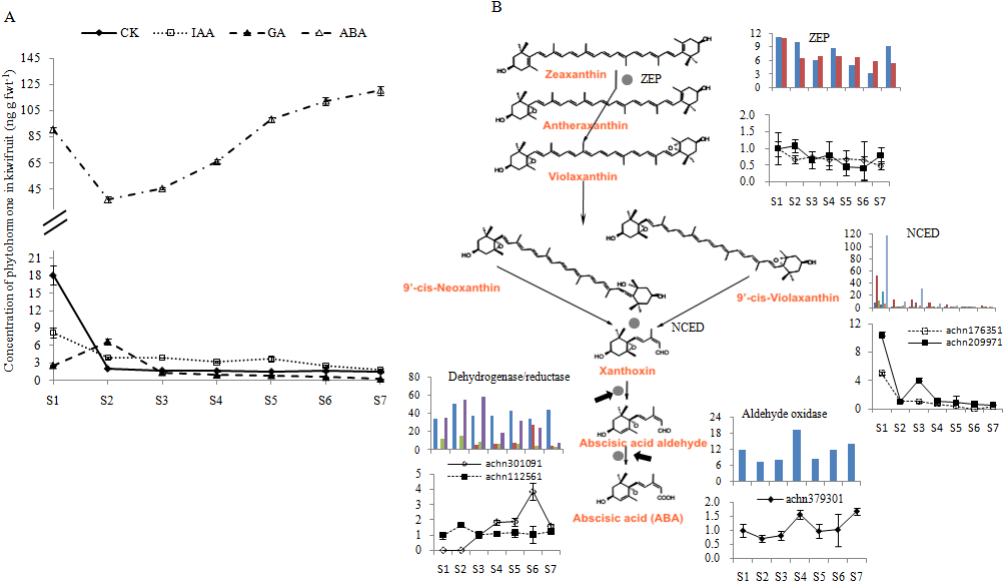
Content of ABA, CK, IAA and GA during *A*. *chinensis* ‘Hongyang' fruit development through HPLC-MS. **A** Phytohormone levels during kiwifruit development. Phytohormone concentration values are means of five replicates. fwt, fresh weight. **B** The transcript levels of some genes committed to ABA biosynthesis. The Y axes of inserted bar graphs are PRKM value from the transcriptome data, and of inserted broken line graphs are the relative expression abundance through qRT-PCR from three biological replicates. ZEP, zeaxanthin epoxidase. NCED, 9-cis-epoxycarotenoid dioxygenases. Error bars represent SE.

Four endogenous phytohormones levels were measured by HPLC and identified through LC-MS (Fig 3 and S3 File). The concentration of ABA in the nascent fruit was at a high level (90.32 ng g fwt^-1^) and, after experiencing a low level during S2 (36.89 ng g fwt^-1^), increased through to the ripening stags (120.36 ng g fwt^-1^). The levels of CK and IAA were higher at S1 stage than at other stages. GA had a lower level at S1, increased to about 6.68 ng g fwt^-1^ at S2, and then remained low through harvest (Fig 3A).

Using MapMan, 431 candidate genes for ABA, CK, IAA and GA biosynthesis, degradation and regulation were identified (Table E in S5 File). The expression levels of most genes were verified through qRT-PCR (Table C in S5 File). Firstly, genes involved in ABA biosynthesis and transduction were explored in detail. For example, two genes encode zeaxanthin epoxidase (ZEP), which exists in the upstream of ABA biosynthesis, showed a stable expression level through the whole developmental stages. Two of the seven identified genes (achn176351 in red and achn209971 in light blue) encode 9-cis-epoxycarotenoid dioxygenases (NCEDs), which convert 9-cis-violaxanthin into xanthoxin, and were highly expressed during S1 and S3, which also be proved by qRT-PCR (Fig 3B). The reductase achn301091 (in red), which oxidizes xanthoxin into abscisic acid aldehyde, was notably increased from S5 to S6, but greatly decreased from S6 to S7, and achn112561 had high level through S1 to S7, which were also consistent with the result from qRT-PCR. The aldehyde oxidase achn379301, which oxidizes abscisic acid aldehyde into ABA, showed higher transcript levels at S7 and S4 (Fig 3B). In ABA signal transduction, such as two abscisic acid receptors, PYL family (achn037141 and achn278311) and PP2C negative regulators (achn167311 and achn050151), remained at high expression levels from S4 to S7. However, most of the genes encoding ABA responsive element binding factors (ABF) had low transcript levels through kiwifruit development (Fig A in S4 File).

The expression levels of cytokinin-related genes across the seven developmental stages are shown in Fig B in S4 File. A large number of genes encoding proteins involved in the synthesis or activation of CKs were highly expressed, especially at late developmental stages, including IPT (cytokinin synthase; achn311071), which catalyzes the formation of isopentenyl-ATP, and cytokinin trans-hydroxylase (CYP735A), for the production of trans-zeatin riboside triphosphate (achn293991). However, zeatin O-xylosyltransferase (ZOX), the last enzyme in the formation of zeatin-O- glucoside, showed higher expression levels during early stages rather than late stages. Many UDP-glycosyl transferases (UGT85A2, UGT85A5 and UGT73C2), which specifically catalyze the transfer of glucose moiety to some atoms or work to protect zeatin against degradative enzymes [41,42], decreased their expression levels at late stages. Some genes necessary for the transduction of CK signals, including CRE1 (histidine kinase 3), AHP (histidine-containing phosphotransfer protein), and response regulators in the B-ARR family, all had low expression levels throughout kiwifruit development.

The expression levels of auxin signaling genes across the seven developmental stages are analyzed through MapMan (Fig C in S4 File). Individual genes in upstream of auxin signaling, including auxin influx carrier (AUX1 family), transport inhibitor response 1 (TIR1), negative receptors auxin-responsive protein (AUX/IAA), and auxin response factor (ARF) steps, showed medium levels. Transcript levels throughout fruit development. A large number of downstream genes encoding AUX/IAA, SAUR, and GH3 proteins, which directly regulate plant growth and development, also had similar expression levels with those of the upstream genes during kiwifruit development [43].

The expression levels of GA-related genes across the seven developmental stages are shown in Fig D in S4 File. There is a subset of genes expressed only during fruit set, S1, with no transcript detected after S3, including three 2-oxoglutarate (2OG) and Fe(II)-dependent oxygenase superfamilies (achn100571, achn128921 and achn304611), three ubiquitin-protein ligases (achn269461, achn162881 and achn269471), and one GA 3-oxidase (GA3ox, achn159721). The biosynthetic genes GA 20-oxidase (GA20ox, red dot), GA3ox (green dots) and GA 2-oxidase (GA2ox, black dots) all were down-regulated with the maturing of kiwifruit. Two CYP701A cytochrome p450 genes, involved in the formation of ent-kaurenoic acid from ent-kaurene, showed decreased expression at late stages (purple dots). The CYP88A gene (brown dot), encoding an ent-kaurenoic aicd hydroxylase, was expressed at a stable, medium level across fruit development. For the signal transduction of gibberellin, the expression levels of all GID1 (orange dots) genes, the first active GA receptor, were high, while six genes, each encoding the negative receptors DELLA (blue dots), were also expressed at stable and high levels.

#### Sugar and starch accumulation

**Fig 4:**
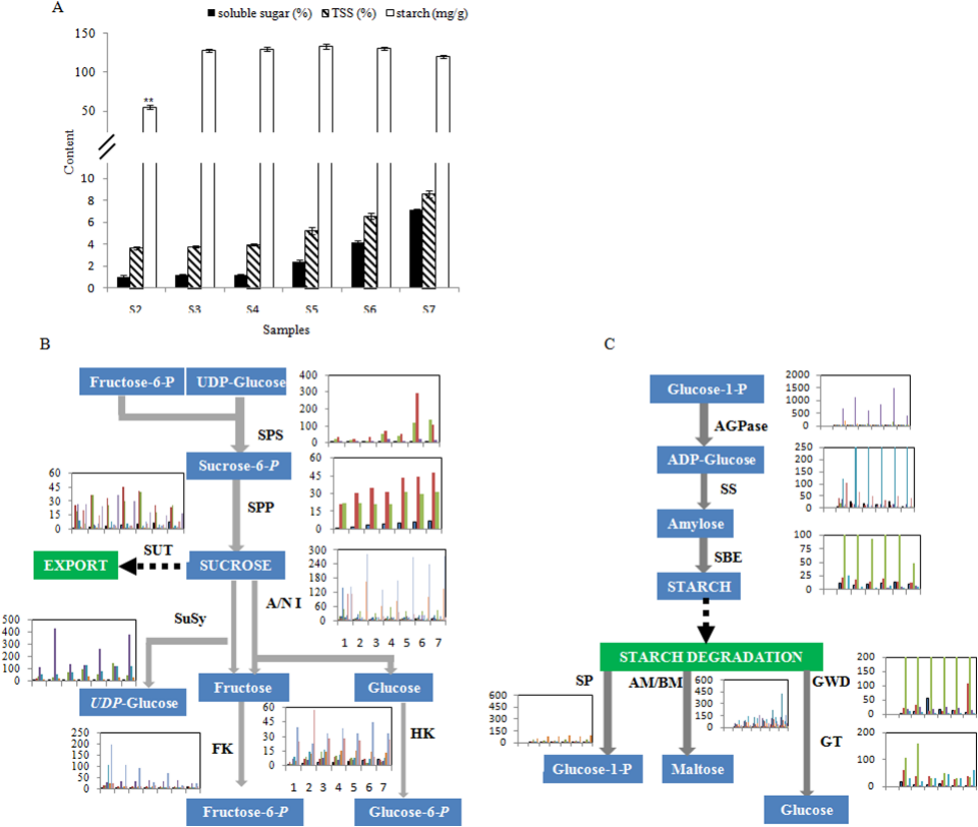
The biosynthesis and metabolism of sucrose and starch in *A*. *chinensis* ‘Hongyang’ during fruit development. (**A)** The content of soluble sugar (% of fwt), starch (mg/g) and total soluble solids (% of fwt) during kiwifruit development. Values are means of ten replicates. Bars indicate ± SE. (**B)** Candidate transcripts putatively involved in the biosynthesis and metabolism of sucrose and **(C)** starch. Inserted bar graphs show expression levels for each candidate gene in any one class across the seven fruit developmental stages, from left (S1) to right (S7), and rows represent each candidate gene. The Y axis represents the transcript value of RPKM. SPS: sucrose-6-P synthase, SPP: sucrose-6F-phosphate phosphohydrolase, SUT: sucrose transporter, SuSy: sucrose synthase, A/NI: sucrose alkaline/ neutral invertase, FK: fructose kinase, HK: Hexokinase, AGPase: ADP-glucose pyrophosphorylase, SS: starch synthase, SBE: starch branching enzyme, SP: starch phosphorylase, AM: α-amylase, BM: β-amylase, GWD: α-glucan water dikinase, GT: 4-α-glucanotransferase. The dashed arrow represents enzymatic steps yet to be identified.

The accumulation of soluble sugars during fruit development largely determines fruit sweetness at harvest. During kiwifruit development, soluble sugars increased from 1.81% in S2 to 7.12% of fwt through in S7 (Fig 4A). Likewise, total soluble solids (TSS) increased from 3.89% to 8.15% of fwt. Starch content increased a large amount from S2 to S3 (57.5 to 129.2 mg g fwt^-1^), stabilized through S6 (about 130 mg g fwt^-1^), and then decreased slightly at ripening (S7, 120 mg g fwt^-1^) (Fig 4A). The expression profiles of most carbohydrate metabolism genes matched the changes in soluble sugar and starch content. For example, the transcript levels for the sucrose synthesis genes SPS (sucrose-6-P synthase), SPP (sucrose-6F-phosphate phosphohydrolase), and SuSy (sucrose synthase) gradually increased during fruit development, however, genes encoding fructose kinase (FK) showed gradually decreasing transcript levels from S1 to S7 (Fig 4B). The transcript levels of A/NI (sucrose alkaline/neutral invertase) and HK (hexokinase) genes, which catalyze sucrose breakdown into glucose and glucose-6-P, were stable. The lower expression levels of HK and FK genes compared with SuSy should result in a low level of fructose-6-P and glucose-6-P. The starch synthesis genes for AGPase (ADP-glucose synthase), SS (starch synthase) and SBE (starch branching enzymes) showed high expression levels during kiwifruit development (Fig 4C), especially the APGases achn372361, which, at 1712 RPKM at S7, was much higher than sucrose biosynthesis genes expression (Fig 4C and 4B). The expression levels of the starch degradation genes *SP* (starch phosphorylase), *AM/BM* (α/β-amylase), *GWD* (α-glucan water dikinase) and *GT* (4-α-glucanotransferase) showed increasing transcript levels as kiwifruit developed, implying the acceleration of starch breakdown at late stages (Fig 4C and Table F in S5 File).

#### L-Ascorbic acid biosynthesis

**Fig 5:**
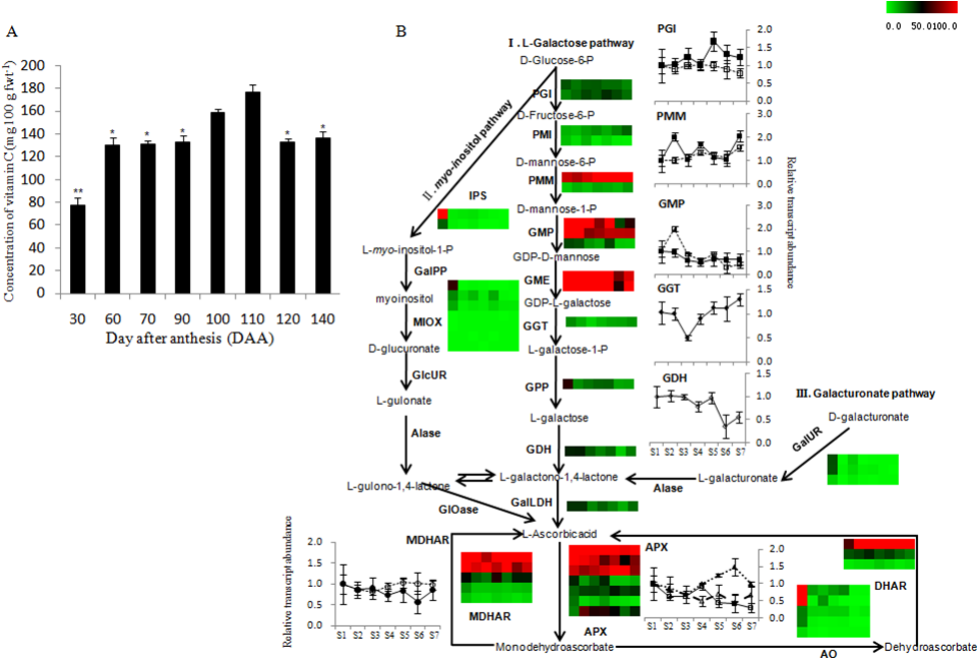
L-Ascorbic acid (vitamin C) biosynthesis and cycling in *A*. *chinensis* ‘Hongyang’ during fruit development. **(A)** Concentration of L-ascorbic acid (vitamin C) during fruit development. Values are means of ten replicates. Bars indicate ± standard error among ten replicates. **(B)** Genes involved in L-ascorbic acid biosynthesis and cycling. Inserted column graphs are from transcriptome data and rows representing each candidate gene. Expression level was represented by a color scale ranging from saturated green for RPKM = 0 to saturated red for RPKM = 100. Inserted broken line graphs are the result of qRT-PCR from three biological replicates. Error bars represent SE. Two *MDHAR* and three *APX* genes with the higher transcript levels were verified. IPS, inositol-3-phosphate synthase; PGI, glucose-6-phosphate isomerase; PMI, mannose-6-phosphate isomerase; PMM, phosphomannomutase; GMP, GDP-D-mannose pyrophosphorylase; GME, GDP-D-mannose- 3,5-epimerase; GGT, GDP-L-galactose phosphate guanylyltransferase; GPP, L-galactose-1-phosphate phosphatase; GDH, L-galactose dehydrogenase; GalLDH, L-galactono-1,4-lactone dehydrogenase; GalUR, D-galacturonic acid reductase; Alase, aldonolactonase; MDHAR, monodehydroascorbate reductase; AO, L-ascorbate oxidase; APX, L-ascorbate peroxidase; DHAR, dehydroascorbate reductase; MIOX, myo-inositol oxygenase.

The level of vitamin C is one of the most important measures of quality in fresh kiwifruit. Vitamin C content in fruit flesh was low at the earliest stage of kiwifruit development (<75 mg 100 g fwt^-1^), reached the highest concentration (174 mg 100 g fwt^-1^) at about 110 DAA (S5), and leveled out at about 137.16 mg 100 g fwt^-1^ at ripening (140 DAA) (Fig 5A). The regulated biosynthesis of L-ascorbic acid (Vitamin C) in kiwifruit was investigated by exploring gene expression levels for three potential pathways: the L-galactose, the galacturonate, and the *myo*-inositol pathways (Fig 5B). It is of interest that the expression levels of genes in the L-galactose pathway, namely those at the top of this pathway encoding PGI (glucose-6-phosphate isomerase) achn087691 increased its expression level at S5, PMM (phosphomannomutase) achn284661 and achn285641 and GMP (GDP-D-mannose pyrophosphorylase) achn055281 and achn258021 were up-regulated in S2. The late genes GGT (GDP-L-galactose phosphate guanylyltransferase) achn339231 showed the lowest level in S3, and GDH (L-galactose dehydrogenase) achn334011 showed lower expression levels in S6, but they all were up-regulated when fruit ripening. While the members of the MIOX family were more highly expressed in kiwifruit than in *Arabidopsis* [44], only three showed high expression at S1 and the others were expressed at low or negligible levels at late stages. The same trend was seen with genes in the galacturonate pathway. Furthermore, the cyclic metabolism of L-ascorbic acid in developing fruit was very active, due to high transcript levels of monodehydroascorbate reductase (MDHAR, achn091771 and achn075231) and L-ascorbate peroxidase (APX, achn082241, achn059971 and achn289741). The phylogenetic analysis of all genes putatively involved in L-ascorbic acid metabolism is shown in S1 Fig and Table G in S5 File.

#### Anthocyanin biosynthesis

**Fig 6:**
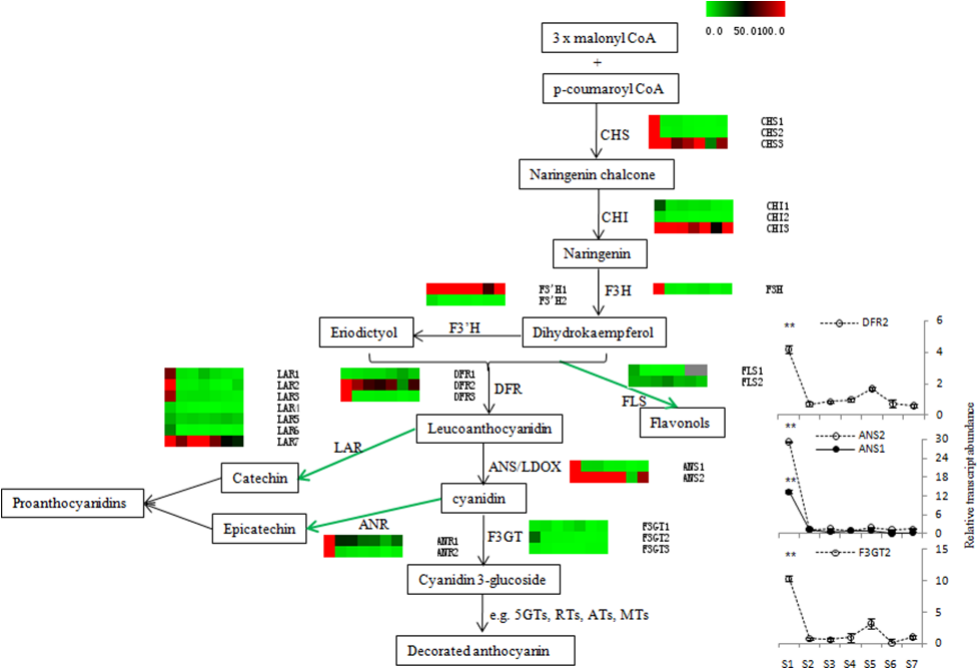
Transcript profiling of genes in flavonoid biosynthesis pathway in *A*. *chinensis* ‘Hongyang’ during fruit development. Each graph is composed of columns for each of the seven developmental stages, from left (S1) to right (S7), and rows representing each candidate gene. CHS: chalcone synthase; CHI: chalcone isomerase; F3H: flavanone 3-hydroxylase; F3’H: flavanoid 3’-hydroxylase; DFR: dihydroflavonol 4-reductase; ANS/LDOX: anthocyanidin synthase/ leucocyanidin oxygenase; F3GT: flavonoid 3-O-glucosyltransferase; FLS: flavonol synthesis; LAR: leucocyanidin reductase; ANR: anthocyanin reductase; 5GTs: 5-glucosyltransferases; RTs: rhamnosyltransferases; ATs: acyltransferases; MTs: methyltransferases. Expression level was represented by a color scale ranging from saturated green for RPKM = 0 to saturated red for RPKM = 100. Insert broken line graphs are the verification through qRT-PCR from three biological replicates. Error bars represent SE. Asterisks indicate significantly different (**P<0.01) from value at 110 DAA (S5).

An appealingly distinct characteristic of ‘Hongyang’ kiwifruit is the anthocyanin accumulation in the flesh. Using 85 flavonoid biosynthetic genes from other plant species, a phylogenetic analysis was conducted against the Kiwifruit Genome (Table H in S5 File and S2 Fig) followed by examination of the transcript levels of these putative anthocyanin genes. During kiwifruit development, the genes *CHS3*, *CHI3*, *F3’H1*, *DFR2* and *ANS2* showed higher expression levels than other putative anthocyanin genes (Fig 6). Genes (*ANS1*, *ANS2*, *DFR2* and *F3GT2*) determining the last steps of anthocyanin biosynthesis were verified through qRT-PCR. Furthermore the expression level of DFR2 and F3GT2 decreased at S6 compared to S5, which was consistent with the decreased anthocyanin level at fruit ripening (Figs 6 and 1B). At absolute levels, the genes in the flavonol and proanthocyanidin biosynthesis pathways were more highly expressed than those leading to anthocyanins, with the expression levels of *LAR7*, *ANR1*and *FLS1* higher compared to that of *F3GT1*. Additionally, the *LAR* gene familiy was large in kiwifruit. Kiwifruit is usually rich in total phenolic acids such as catechin, chlorogenic acid, rutin, epicatechin and quercetin, from 1.8–2.2 mg g fwt^-1^ one month after anthesis to about 1 mg g fwt^-1^ at ripening, levels that are higher than that of anthocyanins [45,46].

**Fig 7:**
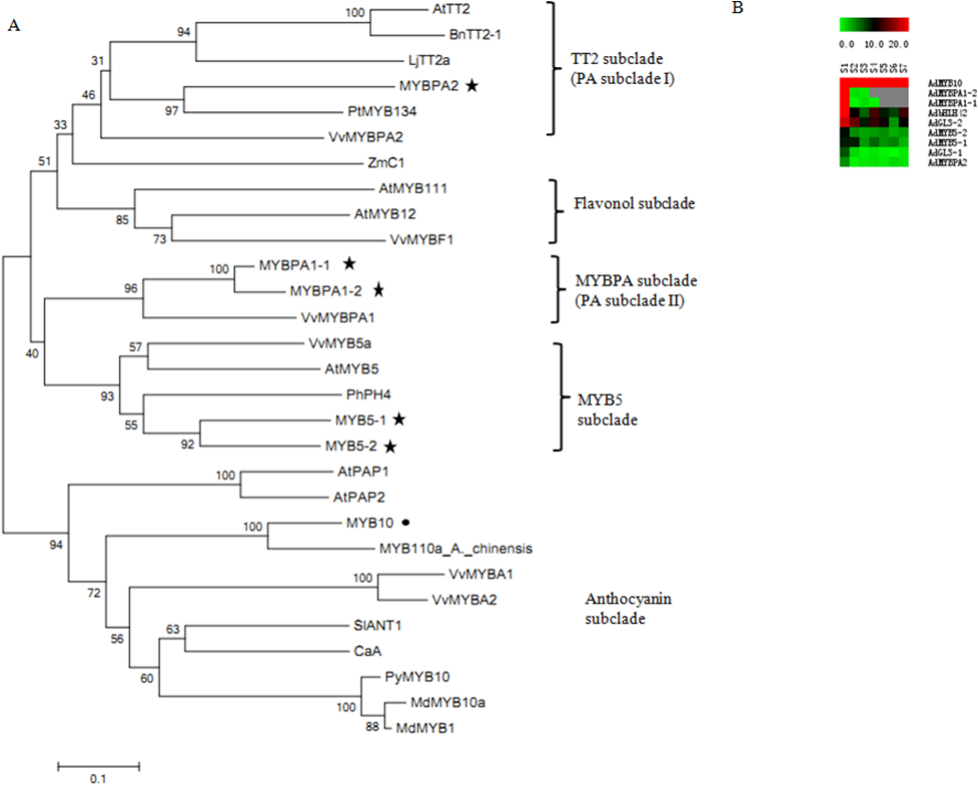
Phylogenetic analysis of the flavonoid-related R2R3-MYB transcription factor family. **(A)** The putative kiwifruit flavonoid regulators identified in ‘Hongyang’ kiwifruit. Trimmed protein sequences were aligned with ClustalW and the linearized neighbor-joining tree was produced with MEGA software version 5.0. GenBank accession number are as follows: BnTT2-1 (*Brassica napus*; ABI13034), AtTT2 (*Arabidopsis*; Q9FJA2), LjTT2a (*Lotus japonicus*; BAG12893), VvMYBPA2 (*Vitis vinifera*, ACK56131), PtMYB134 (*Populus tremuloides*; ACR83705), AtMYB5 (*Arabidopsis*; NP_187963), VvMYB5a (*Vitis vinifera*, AAS68190), PhPH4 (*Petunia*; AAY51377), VvMYBPA1 (*Vitis vinifera*; CAJ90831), AtPAP1 (*Arabidopsis*; AAG42001), AtPAP2 (*Arabidopsis*; NP_176813), SlANT1 (*Solanum lycopersicum*; AAQ55181), ZmC1 (*Zea mays*; AAK09327), AtMYB111 (*Arabidopsis*; NP_199744), VvMYBF1 (*Vitis vinifera*; ACT88298), AtMYB12 (*Arabidopsis*; NP_182268), MdMYB10 (*Malus domestica*; ABB84753), MdMYB1 (*Malus domestica*; ABK58136), VvMYBA1 (*Vitis vinifera*; BAD18977), VvMYBA2 (*Vitis vinifera*; BAD18978), CaA (*Capsicum annuum*; CAE75745), PyMYB10 (*Pyrus pyrifolia*; ADN26574). Genes marked by star from Kiwifruit Genome data and by solid circle from our transcriptome data. **(B)** Expression graph composed of columns for each of the seven developmental stages, from left (S1) to right (S7), and rows representing each candidate transcription factor gene. Expression level was represented by a color scale ranging from saturated green for RPKM = 0 to saturated red for RPKM = 20.

We also used phylogenetic analysis to identify 9 transcription factors potentially involved in anthocyanin metabolism from our transcriptome data (Table H in S5 File). These R2R3 MYB-type transcription factors grouped within other defined subclades containing orthologs with known roles (Fig 7A). *MYB10* clustered with the characterized anthocyanin specific transcription factors including *PAP1* [47], *PyMYB10* [48], *MdMYB10* [49], and *MYB110a* that determined red petal color in kiwifruit [20]. With the exception of *MYB10*, the transcript levels of the other MYB transcription factors were low, especially from S2 to S7 (Top right in Fig 7B). The phylogenetic analysis of other flavonoid biosynthesis-related transcription factors is shown in S3 Fig. *bHLH42* gene was clustered with *DkMYC1*and *AtbHLH42* (*AtTT8*), which regulate proanthocyanidin biosynthesis in persimmon [50] and Arabidopsis [51]. *GL3-2* was also grouped with *AtGL3*, which regulates anthocyanin biosynthesis [52].

**Fig 8:**
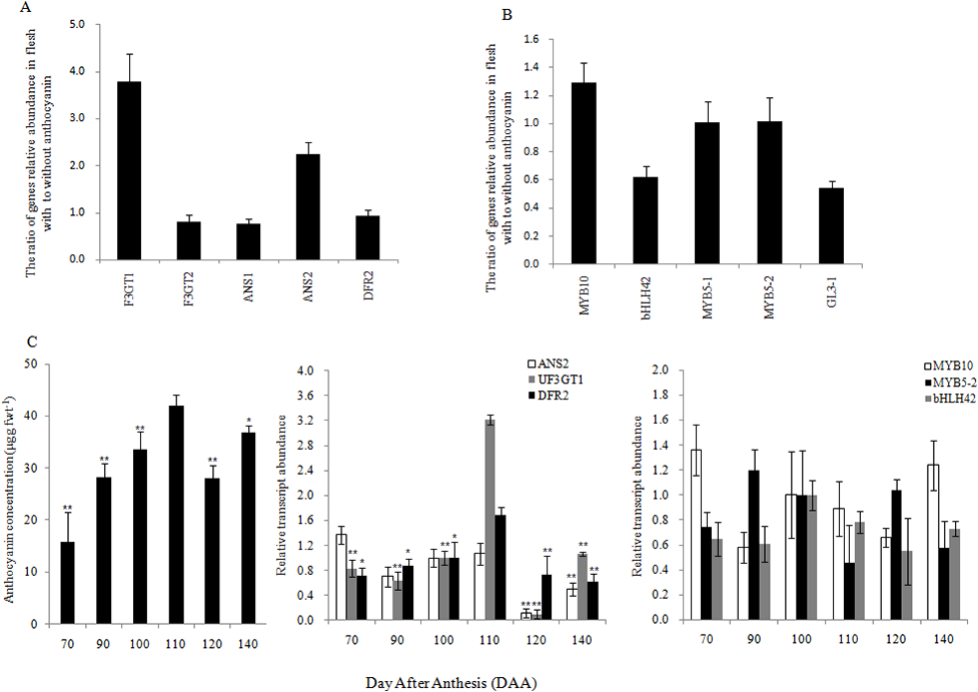
Verification of expression levels for candidate anthocyanin biosynthesis genes. The ratio of biosynthetic genes (A) and transcription factors (B) relative abundance in flesh with to without anthocyanins at 100 DAA. Relationship between anthocyanin concentration and gene expression levels during two continuous years in the orchard **(C)**. The expression levels of all genes were from the red fleshy part (with anthocyanin) and the expression level for each gene at 100 DAA was the calibrator (set to 1). Values of the mean anthocyanin concentration from 20 kiwifruits, and gene relative abundance are means of three biological replicates. Bars indicate ± standard error. Asterisks indicate significantly different (*P<0.05, **P<0.01) from value at 110 DAA (S5).

Since anthocyanin accumulation is localized closest to the seeds within the fruit, the expression of the above identified anthocyanin biosynthetic genes and transcription factors were further analyzed in ‘Hongyang’ kiwifruit flesh with and without visible anthocyanin. In flesh with anthocyanin, the expression levels of *F3GT1* and *ANS2* were four and two times greater than the levels in flesh without anthocyanin, separately (Fig 8A). The level of *DFR2* was similar in the two flesh types (1:1). *ANS1* and *LAR7* showed slightly higher expression in the flesh lacking visible anthocyanin (Fig 8A). The expression levels of transcription factors, although not strikingly different, did indicate that *MYB10* reached higher levels in flesh with anthocyanins (Fig 8B). Furthermore, two years of orchard experiments verified that the peak expression of *F3GT1* and *ANS2* was paralleled by the anthocyanin accumulation peak, especially for *F3GT1* (Fig 8C). Over the two years, the expression levels of *MYB10*, *MYB3-2* or *bHLH42* appeared slightly higher at 100 DAA, right before anthocyanin levels peak (Fig 8C).

## Discussion

In this study, we performed deep transcriptomic surveys in kiwifruit flesh through seven transcriptomic data during kiwifruit development. This is the first set of transcriptomic data to provide genetic and molecular data on kiwifruit development in any cultivar. The detailed mapping of the transcriptional differences through kiwifruit development adds valuable data to the kiwifruit molecular resources and identifies the candidate genes responsible for sought after qualities of kiwifruit, including vitamin C accumulation. Furthermore this analysis has revealed candidate genes responsible for the novel red kiwifruit in ‘Hongyang’. Additionally, genome annotation can be improved through transcript and genome alignment. Numerous novel and alternatively spliced transcripts were identified in addition to what was publicly available in the Kiwifruit Genome database. In the future, AS patterns in different developmental stages and NTs should be validated through more specific experiments, which may indicate some of the AS are related to the development of kiwifruit.

Analysis of physiology (Fig 1A), differentially expressed genes (DEGs, Table 2 and Fig 2), and metabolisms (Figs 3, 4, 5 & 6) indicates that ‘Hongyang’ approaches maturity at about 110 day after anthesis. Interestingly, there was a balance between the numbers of up- and down-regulated genes until the number of down-regulated genes surpassed the number of up-regulated genes at 110 days (Table 2). Abscisic acid and gibberellins are indispensable during the rapid enlargement of fruit, and cytokinin and auxin also play important roles throughout fruit development [53]. We identified many key candidate genes related to phytohormones biosynthesis and signal transduction. ABA may be more important in kiwifruit development due to the high level of both ABA content and expression of ABA-related genes. The levels of the other three phytohormones were low, so it was hard to construct any relationships between gene transcript and phytohormone levels.

Sugar plays an important role not only in fruit development but also in fruit quality and acts through sugar-hormone cross-talk [53,54]. The total content of soluble sugar in ‘Hongyang’ has been reported to be between 8.97–13.45% of fwt, which is higher than other varieties of kiwifruit in the marketplace [2]. Using known genes involved in sugar metabolism from kiwifruit and other fruits [55,56], an overview of sucrose and starch metabolism and accumulation was constructed for ‘Hongyang’. At the early stage of kiwifruit development, the transcript levels of SuSy, FK and HK were high in order to satisfy the rapid utilization of the imported sucrose for fruit growth. As the fruit continues to grow, the expression levels of these genes decreased (Fig 4B), with an accompanying accumulation of fructose and elevated transcript levels of sugar transporters (SUT) and genes that convert excess carbon into starch. At the late stage of kiwifruit development, sucrose accumulation was enhanced with the elevated expression of SPS genes (achn218701 and achn383801).

Transcriptome analysis of genes committed to L-ascorbic acid biosynthesis revealed that L-ascorbic acid in kiwifruit was mainly synthesized through the L-galactose pathway, which was consistent with a previous report [57]. The recycling and oxidation of L-ascorbic acid was very active throughout the whole development of kiwifruit, as seen in the high expression levels of *APX* and *MDHAR*, and likely contributed to the final high level of L-ascorbic acid in ripe fruit. The expanded MIOX gene family might be related to the multiple functions of MIOX enzymes in plants, such as construction of cell wall and the biosynthesis of sugar alcohol [58].

Stage 5, at 110 DAA may be a special stage in kiwifruit development. Anthocyanin accumulation reached its peak at S5, which coincided with expression peaks for most genes in the light reactions, including in photosystem I and photosystem II (S4 Fig). Photosynthesis is the decisive factor in the synthesis and transportation of sugar and bridges the environmental and biological factors that regulate fruit development, including the biosynthesis of anthocyanin [59,60]. However, the photorespiration enzyme GOX (glycolate oxidase) achn176551, which has a reported negative effect on anthocyanin accumulation (14), was highly expressed at S6, which was consistent with the decreased anthocyanin accumulation at S6 [61]. Sugars also play an important role in the expression of anthocyanin biosynthetic genes [62]. For example, the lower expression of sucrose transporter (SUT) at S6 should inhibit the transport of sucrose, which might contribute to the decline in anthocyanin from S5 to S6 [63]. In our study, *F3GT1* showed a potential regulation on ‘Hongyang’ fruit color [19]. As reported, *MYB10* and *MYB110a* were all detected in red but barely in white petals of kiwifruit, and the expression level of *MYB10* was much lower than *MYB110a* [20]. However, MYB10 in ‘Hongyang’ fruit showed a high transcript level, and MYB110a was not included in our transcriptome data. The function of MYB10 on regulation of fruit color should be verified further. Although the precise regulation of anthocyanin, vitamin C and sugar accumulation remains obscure, our analysis of the red kiwifruit transcriptome has identified numerous candidate genes and regulatory connections between the pathways that lead to desirable characteristics in this unique fruit.

## Supporting Information

S1 Fig Phylogenetic analysis of genes involved in L-ascorbic acid biosynthesis and metabolism. (TIF)

S2 Fig Phylogenetic analysis of biosynthetic genes CHS, CHI, F3H, FLS, DFR, ANS, ANR, LAR and F3GT. (TIF)

S3 Fig Phylogenetic analysis of the bHLH and GL3 protein family, involved in flavonoid biosynthesis, based on transcriptome sequencing. (TIF)

S4 Fig The metabolism of photosynthesis throughout ‘Hongyang’ development based on MapMan. (TIF)

S1 File The quality of sequencing and alignment from seven kiwifruit transcirptomic data. Distributions of reads on reference genes of sample **(Fig A).** Pia chart representing gene coverage distribution **(Fig B).**
(TIF)

S2 File Expression level of DEGs involved in metabolism through MapMan software. Metabolism overview of DEGs at S2 compared to S1 (**Fig A**). metabolism overview of DEGs at S6 compared to S7(**Fig B**).
(TIF)

S3 File MS identification of four hpytohormones in ‘Hongyang’ kiwifruit. The chromatographic peaks of four phytohormones in ‘Hongyang’ kiwifruit (Fig A). MS identification of four phytohormones in ‘H ongyang’ kiwifruit (Fig B), in which A represents GA (Gibberellic acid), MW, 346.37; B for IAA (3-indoleacetic acid), MW, 175.18; C for ABA ((±)-Abscisic acid), MW, 264.32; D for CK (trans-zeatin), MW, 219.28. SS, respectively.
(TIF)

S4 File Hierarchical cluster analysis of the expression level of genes committed to phytohormone metabolism. Expression levels of genes involved in the transduction of ABA (Fig A), biosynthesis and transduction of CK (Fig B), of AUX (Fig C) and of GA (Fig D)
(TIF)

S5 File Supporting tables. Table A. The expression profiles of all expressed genes in developing fruit of *A*. *chinensis* cv. 'Hongyang'. Table B. List of novel transcripts, alternative splicing and genes extended identified through analysis of the 'Hongyang' transcriptome. Table C. Verification of RNA-seq results by qPCR in *A*. *chinensis* cv. 'Hongyang'. Table D. Differentially expressed genes between two neighboring stages of fruit development in *A*. *chinensis* cv. ‘Hongyang’. Table E. List of genes involved in ABA, CK, AUX and GA3 biosynthesis and signal transduction in developing fruit of *A*. *chinensis* cv. 'Hongyang'. Table F. List of genes involved in biosynthesis and metabolism of sugar and starch in developing fruit of *A*. *chinensis* cv. 'Hongyang'. Table G. List of genes involved in L-ascorbic acid biosynthesis and metabolism in developing fruit of *A*. *chinensis* cv. 'Hongyang'. Table H. List of genes involved in flavonoid biosynthesis and regulation in developing fruit of *A*. *chinensis* cv. 'Hongyang'.
(ZIP)
