# Correction: Gene Expression Profiling of Development and Anthocyanin Accumulation in Kiwifruit (*Actinidia chinensis*) Based on Transcriptome Sequencing

**DOI:** 10.1371/journal.pone.0138743

**Published:** 2015-09-16

**Authors:** Wenbin Li, Yifei Liu, Shaohua Zeng, Gong Xiao, Gan Wang, Ying Wang, Ming Peng, Hongwen Huang

The Excel file is absent from the published S5 File. Please view the complete S5 File here.

S5 File Supporting Tables. Table A. The expression profiles of all expressed genes in developing fruit of *A*. *chinensis* cv. 'Hongyang'. Table B. List of novel transcripts, alternative splicing and genes extended identified through analysis of the 'Hongyang' transcriptome. Table C. Verification of RNA-seq results by qPCR in *A*. *chinensis* cv. 'Hongyang'. Table D. Differentially expressed genes between two neighboring stages of fruit development in *A*. *chinensis* cv. ‘Hongyang’. Table E. List of genes involved in ABA, CK, AUX and GA3 biosynthesis and signal transduction in developing fruit of *A*. *chinensis* cv. 'Hongyang'. Table F. List of genes involved in biosynthesis and metabolism of sugar and starch in developing fruit of *A*. *chinensis* cv. 'Hongyang'. Table G. List of genes involved in L-ascorbic acid biosynthesis and metabolism in developing fruit of *A*. *chinensis* cv. 'Hongyang'. Table H. List of genes involved in flavonoid biosynthesis and regulation in developing fruit of *A*. *chinensis* cv. 'Hongyang'.
(XLS)
