# Supplementary figures and images for: Gene Expression Profiling of Development and Anthocyanin Accumulation in Kiwifruit (Actinidia chinensis) Based on Transcriptome Sequencing
Source: PLoS One. 2015 Aug 24;10(8):e0136439. doi: 10.1371/journal.pone.0136439 (PMC4547809; doi:10.1371/journal.pone.0136439)

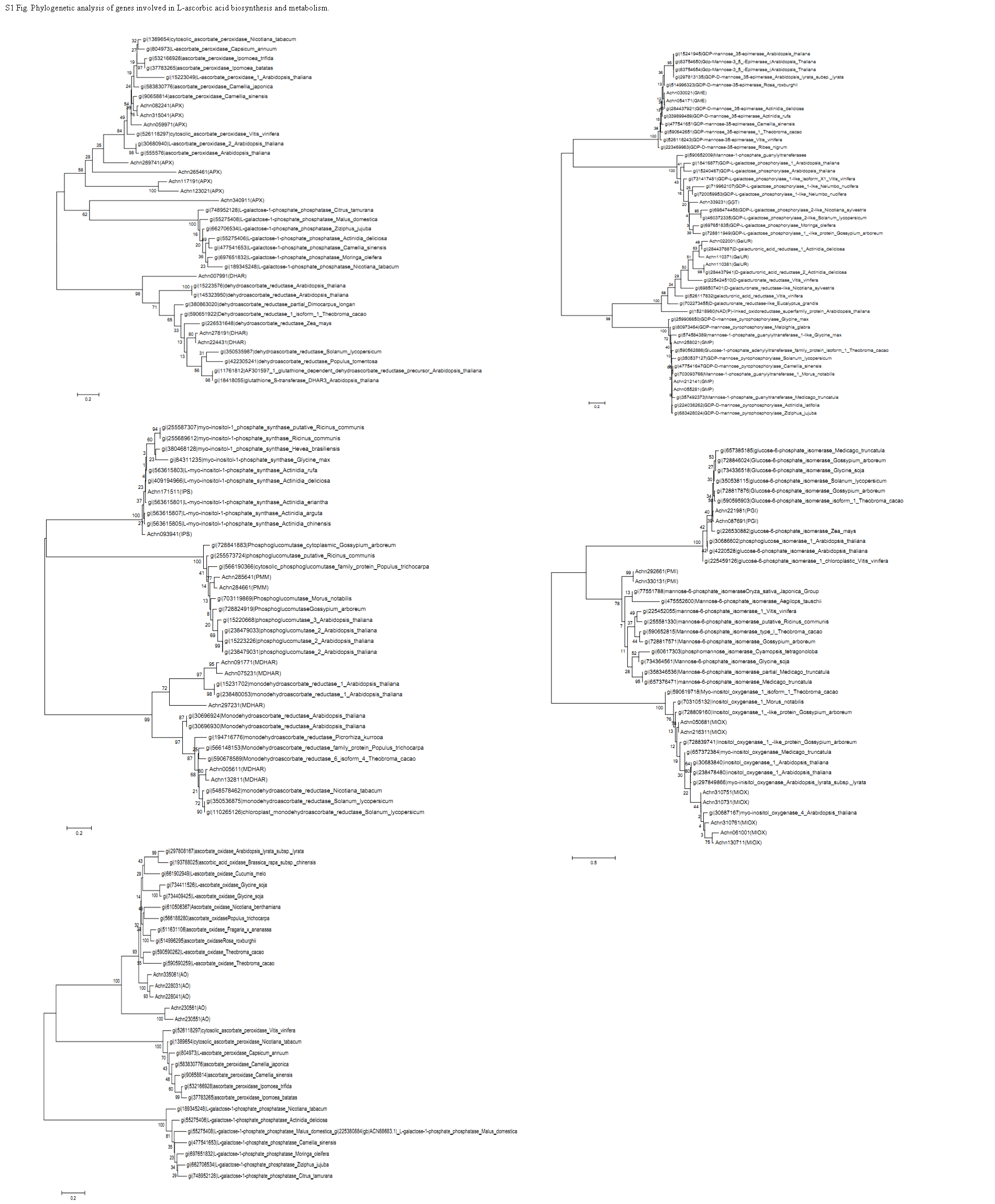

Supplement: S1 Fig — (TIF) [file pone.0136439.s001.tif]

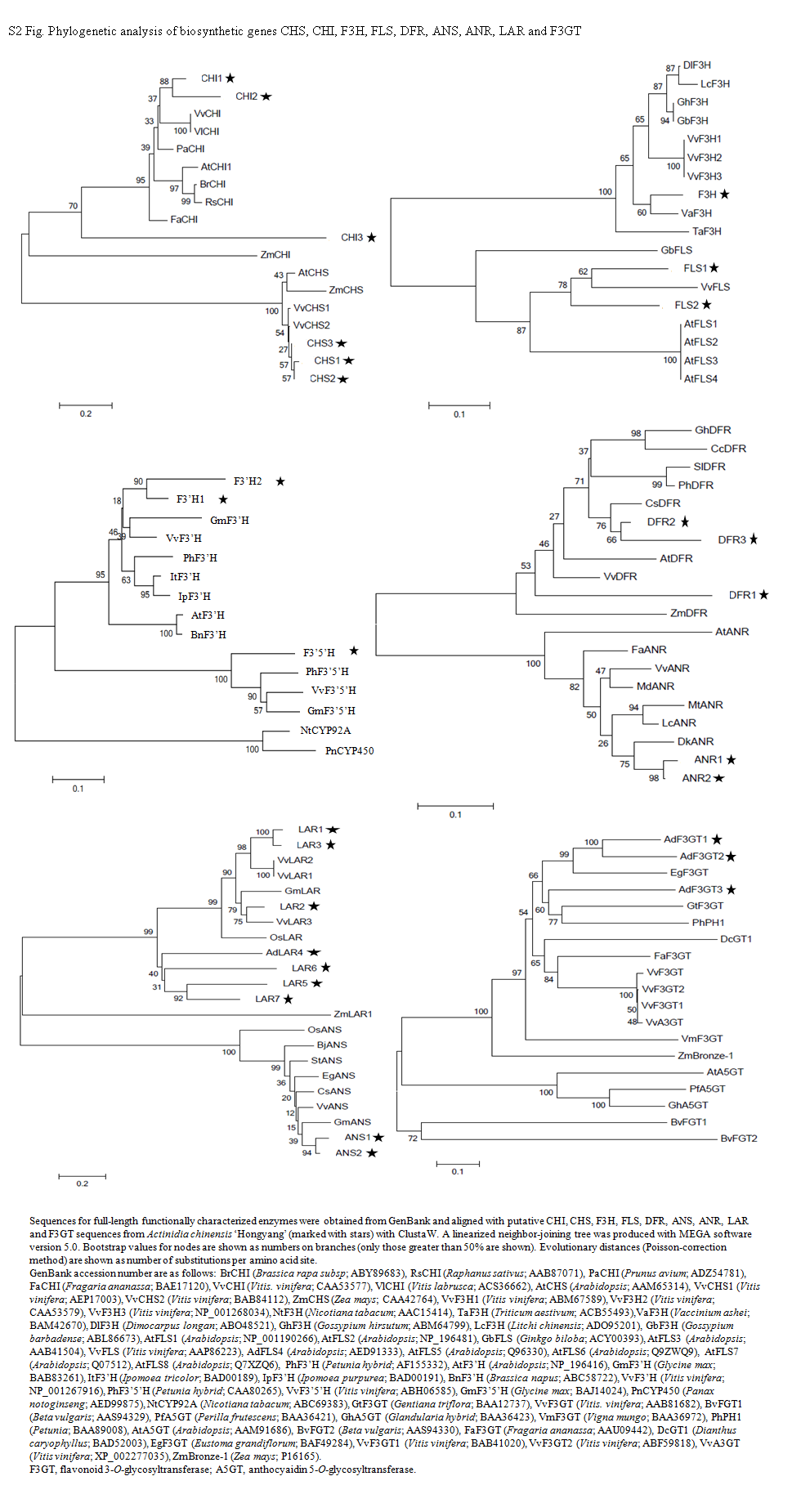

Supplement: S2 Fig — (TIF) [file pone.0136439.s002.tif]

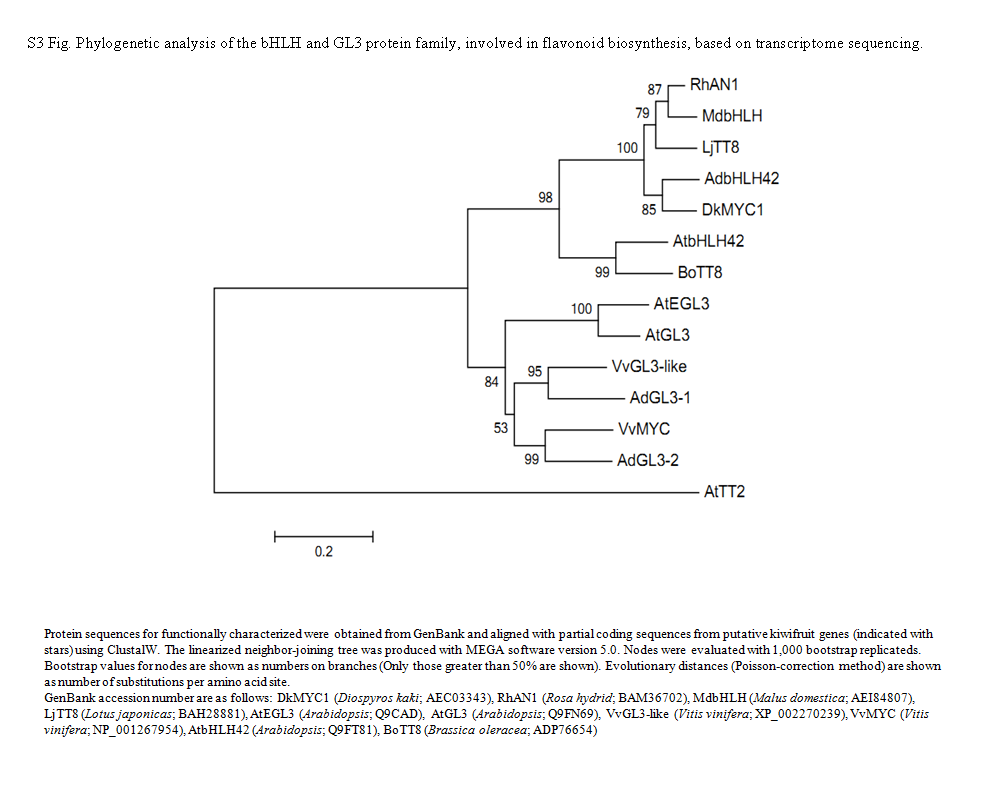

Supplement: S3 Fig — (TIF) [file pone.0136439.s003.tif]

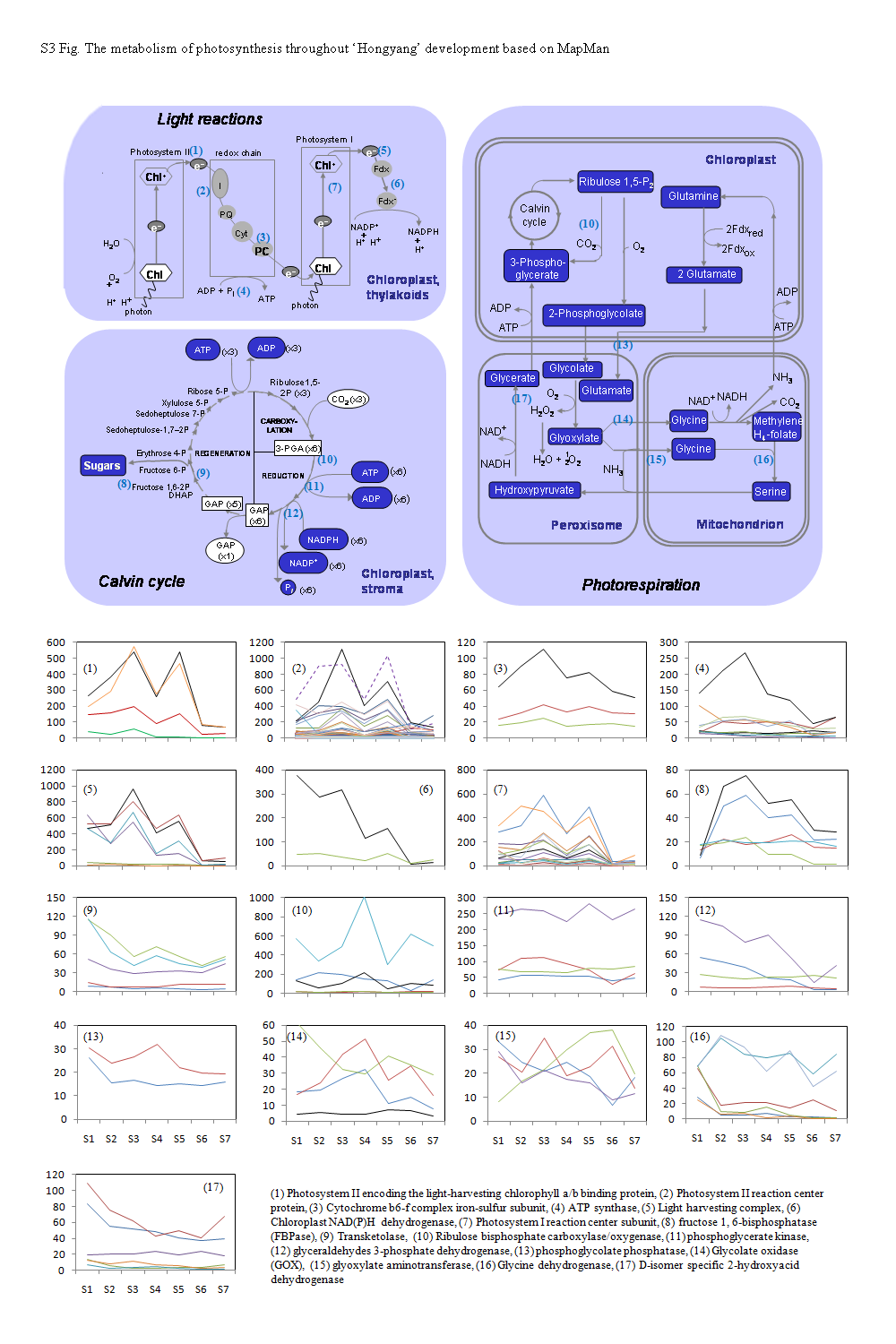

Supplement: S4 Fig — (TIF) [file pone.0136439.s004.tif]

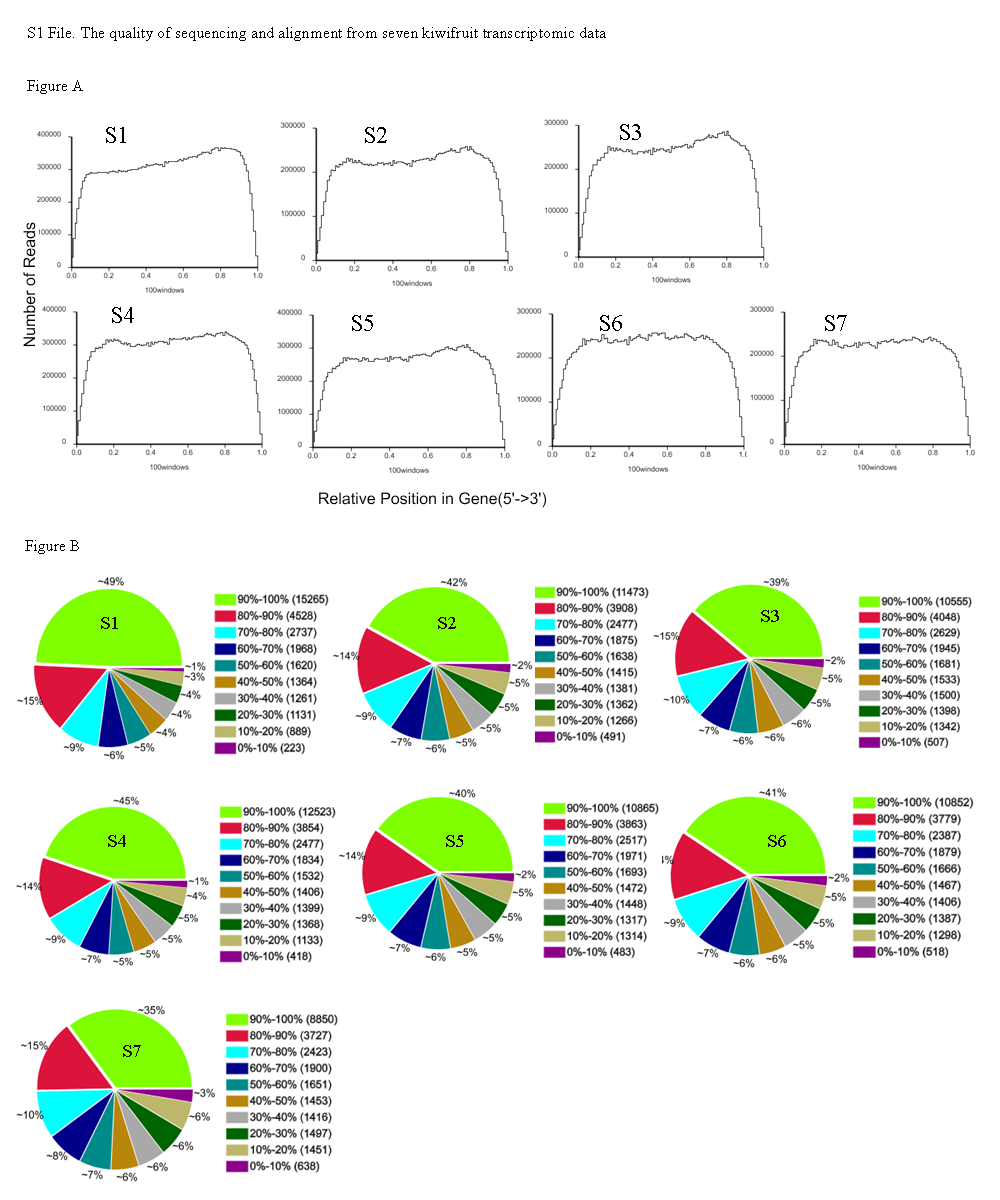

Supplement: S1 File — Distributions of reads on reference genes of sample (Fig A). Pia chart representing gene coverage distribution (Fig B). (TIF) [file pone.0136439.s005.tif]

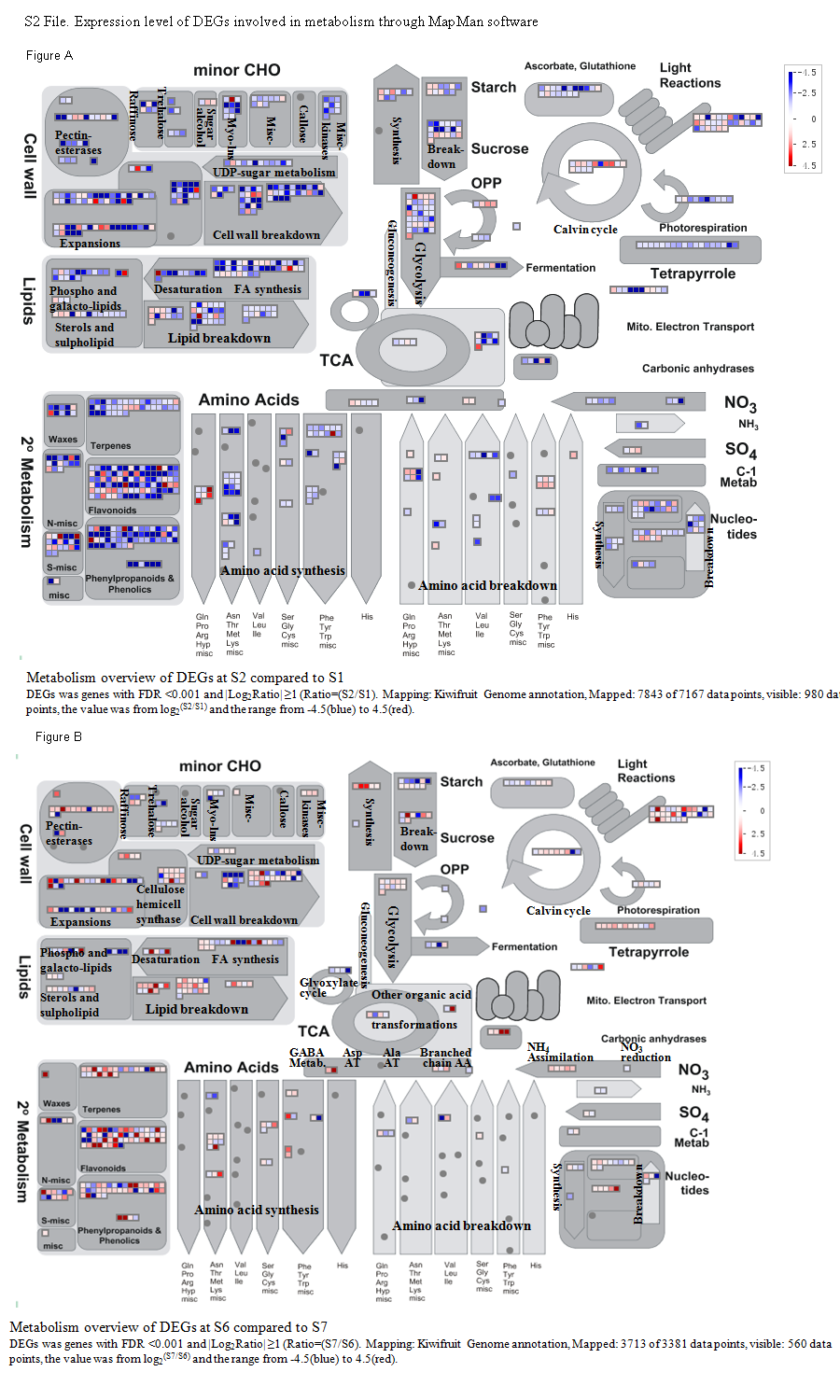

Supplement: S2 File — Metabolism overview of DEGs at S2 compared to S1 (Fig A). metabolism overview of DEGs at S6 compared to S7(Fig B). (TIF) [file pone.0136439.s006.tif]

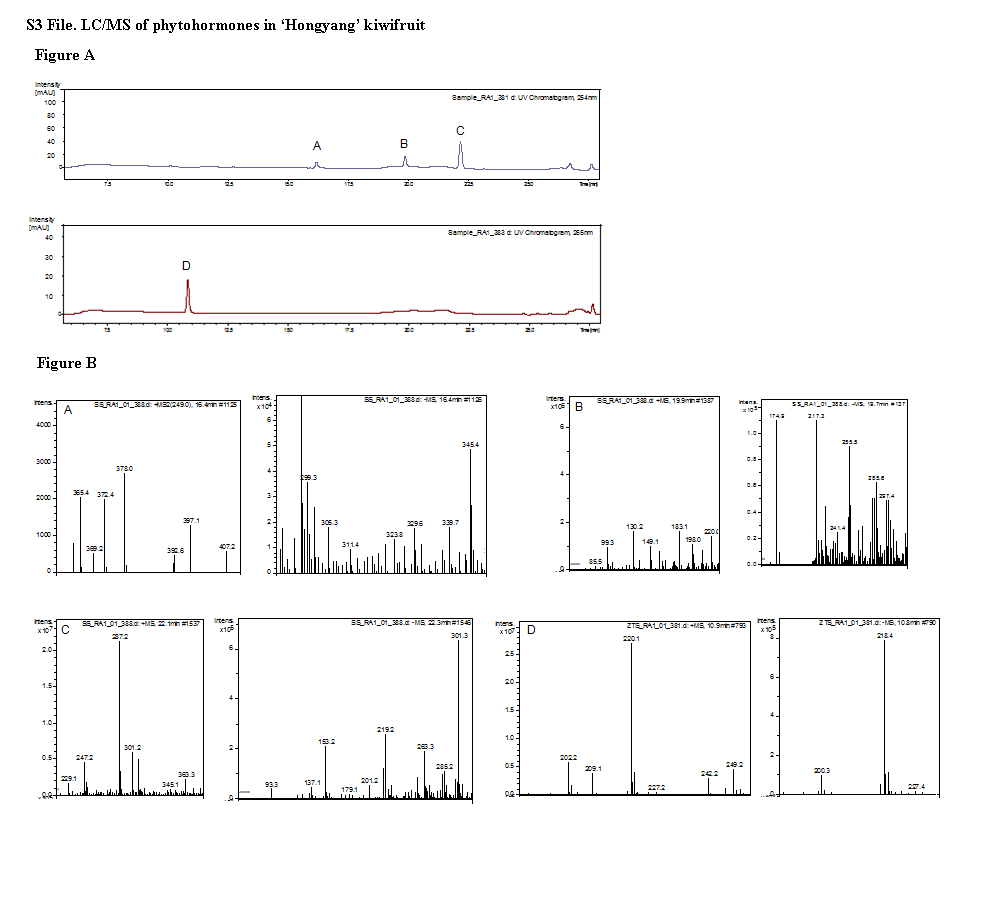

Supplement: S3 File — The chromatographic peaks of four phytohormones in ‘Hongyang’ kiwifruit (Fig A). MS identification of four phytohormones in ‘H ongyang’ kiwifruit (Fig B), in which A represents GA (Gibberellic acid), MW, 346.37; B for IAA (3-indoleacetic acid), MW, 175.18; C for ABA ((±)-Abscisic acid), MW, 264.32; D for CK (trans-zeatin), MW, 219.28. SS, respectively. (TIF) [file pone.0136439.s007.tif]

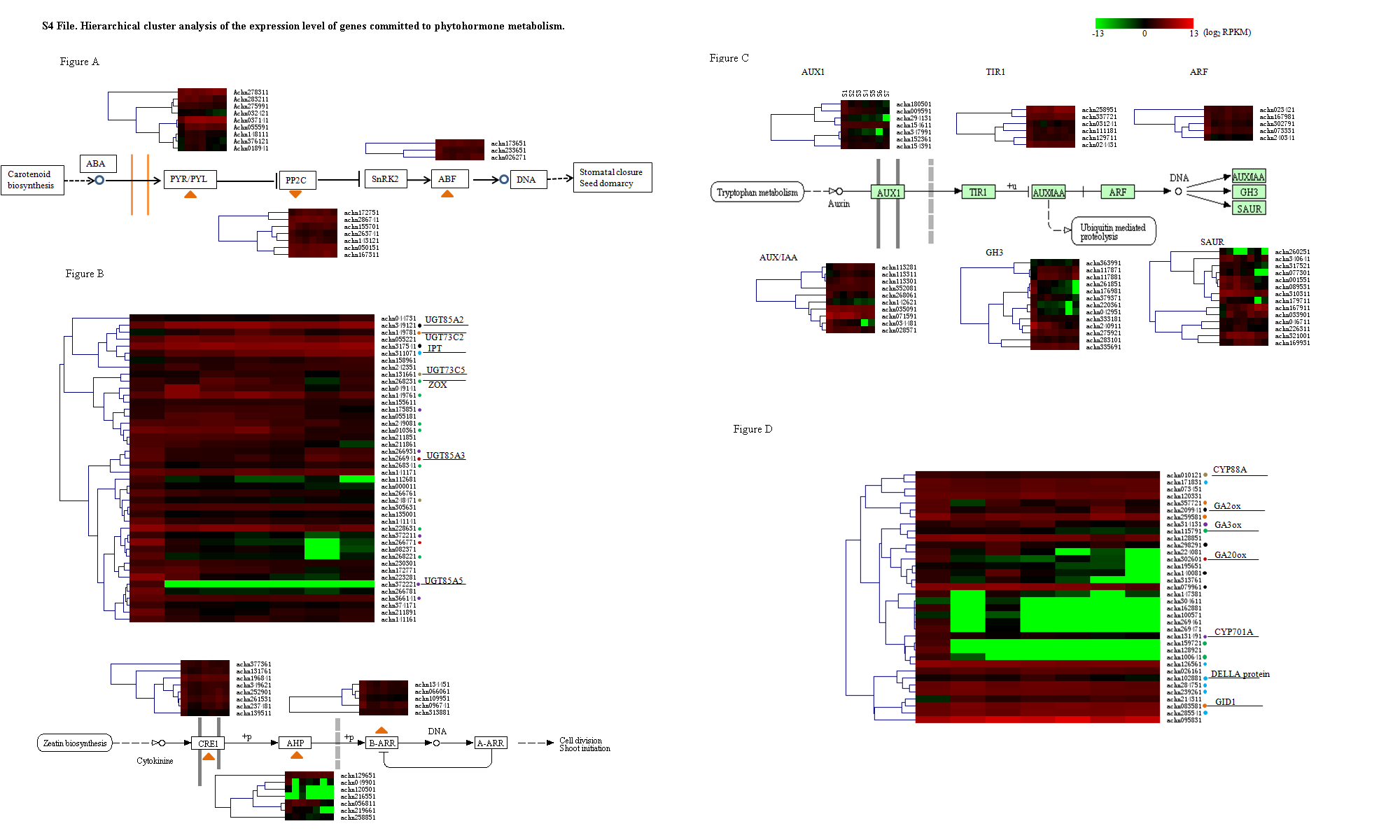

Supplement: S4 File — Expression levels of genes involved in the transduction of ABA (Fig A), biosynthesis and transduction of CK (Fig B), of AUX (Fig C) and of GA (Fig D) (TIF) [file pone.0136439.s008.tif]
